# Clinical Presentations and Outcome Studies of Cranial Nerve Involvement in Herpes Zoster Infection: A Retrospective Single-Center Analysis

**DOI:** 10.3390/jcm9040946

**Published:** 2020-03-30

**Authors:** Po-Wei Tsau, Ming-Feng Liao, Jung-Lung Hsu, Hui-Ching Hsu, Chi-Hao Peng, Yu-Ching Lin, Hung-Chou Kuo, Long-Sun Ro

**Affiliations:** 1 Department of Neurology, Chang Gung Memorial Hospital, 199 Tung Hwa North Road, Taipei 105, Taiwan; william770315@hotmail.com (P.-W.T.); mingfengliao@hotmail.com (M.-F.L.); tulu@ms36.hinet.net (J.-L.H.); kuo0426@adm.cgmh.org.tw (H.-C.K.); 2 Department of Traditional Chinese Medicine, Division of Chinese Acupuncture and Traumatology, Chang Gung Memorial Hospital, Taipei 105, Taiwan; faithjanet@gmail.com; 3 Division of Chinese Internal Medicine, Center for Traditional Chinese Medicine, Chang Gung Memorial Hospital, Taipei 105, Taiwan; toponchu@gmail.com; 4 Department of Medical Imaging and Intervention, Chang Gung Memorial Hospital, Taipei 105, Taiwan; yuching1221@gmail.com

**Keywords:** herpes zoster, cranial nerve, cranial nerve zoster, Ramsay Hunt syndrome

## Abstract

Varicella-zoster virus (VZV) infection can cause chickenpox and herpes zoster. It sometimes involves cranial nerves, and rarely, it can involve multiple cranial nerves. We aimed to study clinical presentations of cranial nerve involvement in herpes zoster infection. We included patients who had the diagnosis of herpes zoster infection and cranial nerve involvement. The diagnosis was confirmed by typical vesicles and a rash. We excluded patients who had cranial neuralgias or neuropathies but without typical skin lesions (zoster sine herpete or post-herpetic neuralgia). We included 330 patients (mean age, 55.0 ± 17.0 years) who had herpes zoster with cranial nerve involvement, including 155 men and 175 women. Most frequently involved cranial nerves were the trigeminal nerve (57.9%), facial nerve (52.1%), and vestibulocochlear nerve (20.0%). Other involved cranial nerves included the glossopharyngeal nerve (0.9%), vagus nerve (0.9%), oculomotor nerve, trochlear nerve, and abducens nerve (each 0.3%, respectively). One hundred and seventy patients (51.5%) had only sensory symptoms/signs; in contrast, 160 patients (48.5%) had both sensory and motor symptoms/signs. Of those 160 patients, sensory preceded motor symptoms/signs in 64 patients (40.0%), sensory and motor symptoms/signs occurred simultaneously in 38 patients (23.8%), and motor preceded sensory symptoms/signs in 20 patients (12.5%). At one month after herpes zoster infection, vesicles and rash disappeared in 92.6% of patients; meanwhile facial palsy showed a significant improvement in 81.4% of patients (*p* < 0.05). Cranial motor neuropathies are not infrequent in herpes zoster infections. Multiple cranial nerve involvement frequently occurred in Ramsay Hunt syndrome. We found a significantly increased seasonal occurrence of cranial nerve zoster in spring rather than summer. Cranial motor nerves were affected while the hosts sometimes had a compromised immune system.

## 1. Introduction

Varicella-zoster virus (VZV) can cause chickenpox as a primary infection. VZV also establishes latent infection in sensory ganglia cells in all individuals who experience primary infection. Subsequent reactivation of the latent virus causes herpes zoster, a painful vesicular rash that usually is in the dermatomes [1,2,3]. Zoster lesions occur most frequently in the trunk but may be seen in any of the dermatomes, including the face [4]. Zoster can also, but does not commonly, occur without a rash (zoster sine herpete), including in the cranial dermatomes. Rarely, herpes zoster manifests as cranial motor neuropathy, including most of the cranial nerves, which most frequent occurred in Ramsay Hunt syndrome (RHS) [5,6]. There were only a few case reports other than RHS documented in the literature [5,6,7,8,9]. Moreover, there were no systematic studies of cranial neuropathy in herpes zoster infection in terms of sensory and motor involvement in the literature. Thus, we aimed to study the clinical presentations and outcome of patients with cranial nerve zoster.

## 2. Material and Methods

The study involved a medical chart review of patients with cranial nerve zoster. The study protocol was approved by the institutional review board of Chang Gang Memorial Hospital (serial number: 201900143B0).

We retrospectively reviewed the medical records of the patients who had the diagnosis of herpes zoster infection and cranial nerve involvement from January 2008 to December 2017. The diagnosis was confirmed by typical vesicles and rash. Those who had post-herpetic neuralgia but had no typical skin lesions were excluded. The patients with acute cranial neuropathies due to potential zoster without a rash were also excluded. We recorded the age, gender, comorbidities, clinical symptoms/signs, cranial nerve involvements, treatment, and the one-month outcome in follow-up patients. As a surrogate for the recovery of sensory nerve involvement, we recorded the disappearance of the vesicles and rash. For the improvement of motor nerves (facial palsies), we used the House-Brackmann (HB) facial nerve grading system [10]. We defined the significant improvement of facial palsy as patients with initial HB grades III to VI (moderate to total paralysis) who improved to HB grades I or II (normal/mild dysfunction), or patients with initial HB grade II who improved to grade I. We evaluated the recovery ratios at one month after onset of herpes zoster infection in all follow-up patients.

Statistical analyses were performed using a statistical software package (IBM SPSS Statistics Subscription; IBM, New York, NY, USA). A Mann–Whitney U test, *z* test, *t* test, Chi-Square, or Fisher’s exact test were used when appropriate. A *p* value < 0.05 was considered statistically significant.

## 3. Results

**Figure 1:**
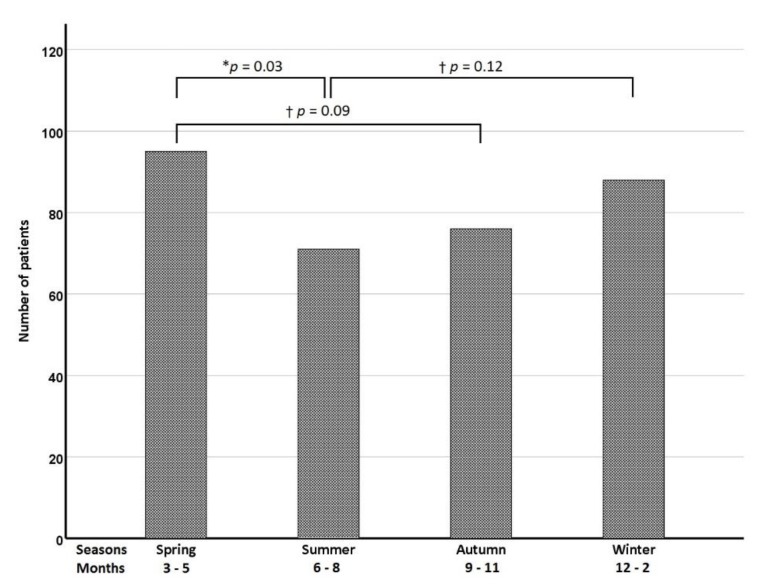
Seasonal and monthly distributions in patients with cranial nerve zoster. * *p* *<* 0.05, *z* test; † *p >* 0.05, *z* test.

A total of 330 patients (mean age, 55.0 ± 17.0 years) with herpes zoster infection and cranial nerve involvement were identified in this study. Table 1 shows the demographic data and clinical information for the study population. One fourth of the patients had comorbidities with diabetes mellitus (14.8%) which was the leading associated disease, followed by malignancy (5.2%). Cranial nerve zoster was also found in other immune-compromised patients such as autoimmune disease (3.0%) or end stage renal disease (1.5%). Figure 1 shows seasonal distributions in patients with cranial nerve zoster. There is a significantly increased seasonal occurrence of cranial nerve zoster in spring rather than summer (*p <* 0.05).

The most frequently affected cranial nerves in VZV were the trigeminal nerve (57.9%), facial nerve (52.1%), and vestibulocochlear nerve (20.0%). Other VZV-affected cranial nerves including the glossopharyngeal nerve (0.9%), vagus nerve (0.9%), oculomotor nerve (0.3%), trochlear nerve (0.3%) and abducens nerve (0.3%) rarely occurred (Table 1).

Herpes zoster ophthalmicus is defined as herpes zoster involvement of the ophthalmic division of the trigeminal nerve. It is the most common and most troublesome [4]. Multiple trigeminal branches could be affected in 8.9 percent of our patients with VZV trigeminal neuropathy (Table 2).

Interestingly, all multiple cranial nerve involvement in VZV were associated with RHS. Table 2 lists the involvement of these multiple cranial nerves in this study. The most frequent affected cranial nerve associated with RHS was vestibulocochlear nerve, followed by the trigeminal nerve. Occasionally, RHS complicated with the glossopharyngeal and vagus nerves. Rarely, one patient with zoster in trigeminal ophthalmic branch developed complete ophthalmoplegia affecting the oculomotor, trochlear, and abducens nerves.

The clinical manifestations of herpes zoster are usually rash and pain. However, near a half of patients also had cranial motor neuropathies (Table 3). Almost all cranial motor neuropathies were associated with RHS (facial palsy and external auditory canal vesicles and a rash). Analysis of the time sequence for the occurrence of sensory symptoms/signs or motor neuropathies revealed that, in only 12.5 percent of patients, motor neuropathy preceded the sensory symptoms/signs (Table 3).

The median duration for the disappearance of vesicles and rash was 11.0 days (range: 3 to 58) but for a significant improvement of facial palsy it was 19.0 days (range: 4 to 194, *p <* 0.001). Table 4 shows the outcomes at 1 month after the onset of herpes zoster infection in those follow-up patients. The recovery ratio between sensory symptoms/signs and motor neuropathies was also significantly different (92.6% vs. 81.4%, *p* < 0.05). Facial palsy took a longer time to recover in this study. One patient was reported to begin to show a significant improvement of his facial palsy at 194 days after the onset of herpes zoster infection. Most of our patients received a course of acyclovir treatment (7 to 10 days) and some patients received a short course of steroids (7 to 10 days) for facial palsy. In follow-up patients with facial palsy, there is a tendency that the patients receiving acyclovir plus steroid treatments had a shorter recovery time and a significantly higher recovery ratio as compared to those patients with acyclovir treatment only (*p* < 0.05, Fisher’s exact test, Table 5).

## 4. Discussion

After the initial infection of chickenpox, VZV remains in a latent form in the dorsal root ganglia or cranial nerve ganglia without producing clinical manifestations. Reactivation of this latent virus causes herpes zoster [1,2,3]. Zoster lesions are usually unilateral and occur most frequently in the trunk but may be seen in any of the dermatomes, including the face [4]. In this study, we mainly analyzed the clinical presentations, demographics, seasonal occurrence, and outcomes of cranial nerve zoster.

Unlike chickenpox, previous studies showed no seasonal variations in herpes zoster infection [11,12]. However, our study showed a significantly increased seasonal occurrence of cranial nerve zoster in spring rather than summer (*p* < 0.05). There is one recent Korean study which also demonstrated a seasonal variation, which is comparable to our study [13]. We have no good explanations for the seasonal variations of the occurrence of herpes zoster infection. However, the climate of South Korea (34 to 38 degrees north latitude) in spring (2–10 °C in March to 13–23°C in May) and summer (18–27 °C in June to 22–30 °C in August) is similar to the climate of Taiwan (22 to 25 degrees north latitude) in winter (16–21 °C in December to 14–20 °C in February) and in spring (16–22 °C in March to 22–29 °C in May) [14], which may account for the similar results of the occurrence of herpes zoster infection. In this study, 25 percent of the patients with cranial nerve zoster had comorbidity. Among the comorbidities, diabetes mellitus and malignancy are commonly associated with herpes zoster infection. Most patients with immunocompromised comorbidities usually had more severe or even disseminated zoster [15]. Thus, the findings may raise the importance of the zoster prevention by a modern vaccination [16].

In this study, according to the cranial nerve involvement, zoster occurred most frequently in the distribution of trigeminal and facial nerves. The vestibulocochlear nerve was also commonly affected. All these cranial nerve zoster have their own clinical characteristics. Cranial nerve zoster usually occurs within the dermatome of one, or less commonly two, sensory nerves [1,2,17]. In this study, VZV usually affected a single trigeminal branch, but less commonly two branches of trigeminal nerve could be affected, which is consistent with a previous study [4]. The ophthalmic branch of trigeminal nerve was the most common lesion site in cranial nerve zoster.

**Figure 2:**
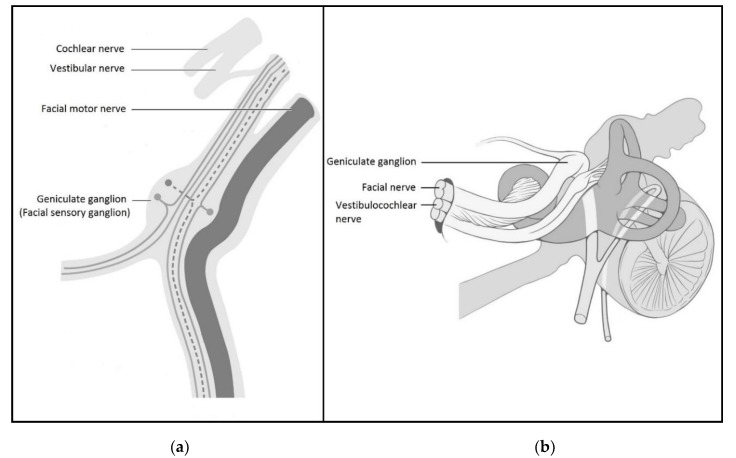
(**a**) A close relationship of the geniculate ganglion to the facial motor nerve. Modified from Duu’s Topical Diagnosis in Neurology 2005, 4th edition, permitted by Thieme Medical Publishers; (**b**) A close relationship of the geniculate ganglion to the vestibulocochlear nerve. Modified from the Creativecommons.org.

Facial nerve zoster is also very characteristic. Nearly all facial nerve lesions in herpes zoster infection (160 of 172 (93.0%) patients in this study) are composed of zoster vesicles in the auricle, external auditory canal and mouth (facial sensory nerve), and facial palsy (facial motor nerve). These characteristics of facial nerve zoster have been defined as Ramsay Hunt syndrome (RHS) [5,6,18]. The close proximity of geniculate ganglion to the facial motor nerve could explain why RHS can affect both facial sensory and motor branches [5,18] (Figure 2a). The most frequently affected motor nerve in cranial nerve zoster is facial nerve involvement. Peitersen [19] reported that 4.5 percent of all peripheral facial palsies were caused by herpes zoster infection. In this study, we found that RHS is the second frequent cranial nerve zoster, which is second to trigeminal nerve zoster.

Herpes zoster infection with multiple cranial nerve involvement often occurred in RHS. In addition to the facial neuropathy manifested as facial palsy and external auditory canal vesicles and rash, vestibulocochlear neuropathy such as tinnitus, hearing loss, and vertigo could also occur [20]. In zoster with multiple cranial nerve involvement, the vestibulocochlear nerve is most commonly involved with the cranial nerve in RHS because of its close proximity to the geniculate ganglion [18,20] (Figure 2b). In this study, the findings of vestibulocochlear neuropathy in zoster with multiple cranial nerve involvement also supports the hypothesis that close proximity of cranial sensory ganglia and motor nerves can contribute to the occurrence of zoster cranial motor neuropathy.

Herpes zoster infection rarely affects cranial motor nerves other than the facial nerve. In this study, we also found that patients with RHS can occasionally affect glossopharyngeal and vagus nerves. Interestingly, one patient had trigeminal ophthalmic zoster associated with oculomotor, trochlear and abducens nerve involvement. These zosters with multiple cranial nerve involvement were rarely reported in the literature [7,8,9]. We raise a hypothesis to explain the occurrence of cranial motor neuropathy in herpes zoster infection. The reactivation of latent VZV in the cranial sensory ganglia (geniculate ganglion and/or trigeminal ganglion) may activate cellular immune responses and induce collateral damage to nearby single or multiple cranial motor nerves due to a close proximity of the cranial motor nerves and sensory ganglia [21].

Zoster is caused by the reactivation of a latent virus from the sensory ganglia. Theoretically, in patients with both sensory and motor symptoms/signs, they should have sensory symptoms preceding, or at least appearing in, both motor and sensory symptoms/signs simultaneously. In this study, most patients with sensory symptoms/signs and motor neuropathies followed the time sequences. However, in 12.5 percent of patients with RHS, their facial palsies preceded the appearance of vesicles and rash. The actual underlying mechanisms still remain unknown. A prospective study of RHS by Murakami et al. [22], reported that 34 percent of their patients developed vesicles and rash after the onset of facial palsies, which is similar to this study.

In RHS, most patients with facial palsies took weeks to months before a significant recovery was seen [18]. In follow-up patients with facial palsy, there is a tendency that the patients receiving acyclovir plus steroid treatments had a shorter recovery time and a significantly higher recovery ratio than those with acyclovir treatment only (*p* < 0.05, Table 5). The number in the group of patients without steroid treatment was very small (*n* = 5), and thus the correlation might be weak. The findings may suggest that zoster cranial motor neuropathy (mainly RHS) might use acyclovir plus steroid treatments to speed up the recovery. The vesicles usually crust in 14 to 21 days [15,18]. In this study, median duration for a significant recovery of the facial palsies was 19.0 days (range: 4 to 194), and median duration for the disappearance of vesicles was 11.0 days (range: 3 to 58). These results suggested that patients with cranial sensory symptoms/signs in herpes zoster infection showed a significantly shorter recovery time than those with cranial motor neuropathy (*p* < 0.05, Table 4), which is consistent with several previous studies [15,18].

Our study has some limitations. First, the sample size of both follow-up groups might have been too small for an adequate comparison. Second, the patient population was from a single referral center, and therefore, may not represent the entire population. Third, the retrospective nature of this study did not allow us to evaluate the actual recovery of follow-up patients in detail. Fourth, the statistical analyses might not be stringent. Last, we did not evaluate zoster sine herpete with cranial nerve involvement because there was lack of virological testing data. Nevertheless, our study provides useful information in cranial nerve zoster. Further prospective studies should be carried out to consolidate these observations.

## 5. Conclusions

In conclusion, we have shown clinical presentations, seasonal variations, and clinical outcomes of cranial nerve zoster. The cranial motor nerves may be affected through reactivation of latent VZV in the adjacent sensory ganglia that can activate cellular immune responses with collateral damages to nearby cranial motor nerve(s). Multiple cranial nerve involvement can sometimes occur in RHS but rarely in trigeminal nerve zoster. This study also suggests that cranial motor neuropathy in herpes zoster infection (mainly RHS) might use acyclovir plus steroid treatments to improve the recovery ratio and shorten the recovery time.

## Figures and Tables

**Table 1:** The demographics in 330 patients with cranial nerve zoster.

Variables		
Age, years	55.0 ± 17.0	
Sex, *n* (%)		
Female	175	(53.0%)
Male	155	(47.0%)
Previously healthy, *n* (%)	247	(74.8%)
Comorbidities, *n* (%)	83	(25.2%)
DM	49	(14.8%)
Malignancy	17	(5.2%)
Autoimmune disease	10	(3.0%)
ESRD	5	(1.5%)
Hematologic disease	3	(0.9%)
Liver cirrhosis	3	(0.9%)
Renal transplant	2	(0.6%)
Cranial nerve involvement, *n* (%)		
CN III	1	(0.3%)
CN IV	1	(0.3%)
CN V	191	(57.9%)
CN VI	1	(0.3%)
CN VII	172	(52.1%)
CN VIII	66	(20.0%)
CN IX	3	(0.9%)
CN X	3	(0.9%)

Symbols and abbreviation: DM = diabetes mellitus; ESRD = end stage renal disease; CN III = oculomotor nerve; CN IV = trochlear nerve; CN V = trigeminal nerve; CN VI = abducens nerve; CN VII = facial nerve; CN VIII = vestibulocochlear nerve; CN IX = glossopharyngeal nerve; CN X = vagus nerve.

**Table 2:** The involvements of trigeminal branches and multiple cranial nerves in 330 patients with herpes zoster infection.

Cranial Nerves	Patients	
	*n* (%)	
Trigeminal branches		
CN V-1	105	(31.8%)
CN V-2	44	(13.3%)
CN V-3	54	(16.4%)
CN V-1, 2	8	(2.4%)
CN V-2, 3	9	(2.7%)
CN V (Unknown)	5	(1.5%)
Involvements of multiple cranial nerves		
CN VII, VIII	71	(21.5%)
CN V, VII	33	(10.0%)
CN V, VII, VIII	9	(2.7%)
CN VII, VIII, IX, X	3	(0.9%)
CN III, IV, V, VI, VII	1	(0.3%)

Symbols and abbreviation: CN V-1 = trigeminal nerve ophthalmic branch; CN V-2 = trigeminal nerve maxillary branch; CN V-3 = trigeminal nerve mandibular branch.

**Table 3:** The involvements of cranial sensory symptoms/signs and motor neuropathies in patients with herpes zoster infection.

Symptoms	Patients
	*n* (%)
Sensory symptoms/signs only	170 (51.5%)
Sensory symptoms/signs and motor neuropathies	160 (48.5%)
Facial palsy	160
Other motor palsy	4
Sensory symptoms/signs precedes motor neuropathies	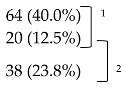
Motor neuropathies precedes sensory symptoms/signs	
Sensory symptoms/signs and motor neuropathies occurred simultaneously	
Unknown	38 (23.8%)

^1^*p* < 0.001, *z* test; ^2^ *p* = 0.009, *z* test.

**Table 4:** Outcome of the cranial sensory and motor symptoms/signs in patients with herpes zoster infection.

Clinical Improvement	Sensory S/S (Vesicles and Rash)	Motor (Facial Palsy)	*p* Value
	(*n* = 108)	(*n* = 59)	
Duration of significant improvement in days			
mean (SD)	14.6 (10.2)	26.2 (32.2)	0.009 ^1^
median (range)	11.0 (3–58)	19.0 (4–194)	< 0.001 ^2^
Improvement ratios at 1-month follow-up, *n* (%)	100 (92.6%)	48 (81.4%)	0.029 ^3^

^1^ by *t* test; ^2^ by Mann-Whitney U test; ^3^ by *z* test; SD = standard deviation; S/S = symptoms and signs.

**Table 5:** Outcome of the facial palsy in patients with herpes zoster infection.

Treatment	Outcome	*p* Value
One-month recovery ratios (%)		
Acyclovir(+) Steroid(−) (*n* = 5)	40.0% (2/5)	
Acyclovir(+) Steroid(+) (*n* = 43)	88.4% (38/43)	0.027 ^1^
Median recovery duration in days median (range)		
Acyclovir(+) Steroid(−) (*n* = 5)	35.0 (22–168)	
Acyclovir(+) Steroid(+) (*n* = 43)	18.0 (4–194)	0.306 ^2^

^1^ by Fisher’s exact test; ^2^ by *t* test.
